# Improving Diagnostic Quality of Anogenital Photodocumentation in Emergency Department Evaluations for Acute Child Sexual Assault

**DOI:** 10.1097/pq9.0000000000000876

**Published:** 2026-04-06

**Authors:** Zachary E. Miller, Sara J. Digirolamo, Jennifer Molnar, Colleen E. Bennett, Sabrina Darwiche, Brandon C. Ku, Philip V. Scribano

**Affiliations:** From the * Department of Pediatrics, Virtua Health College of Medicine & Life Sciences of Rowan University, Stratford, N.J.; † Department of Pediatrics, Children’s Hospital of Philadelphia, Philadelphia, Pa.

## Abstract

**Introduction:**

Emergency department evaluations for acute child sexual assault include anogenital photodocumentation as a standard of care, which has implications for accurate clinical care and forensic significance. If photodocumentation quality is suboptimal from a diagnostic standpoint, this may lead to wasted care and resources and place needless burdens on patients and families. Our project aimed to improve the diagnostic quality of anogenital photodocumentation at 2 sites in a pediatric emergency department, increasing it from a baseline of 24% to 50% during a 9-month intervention period.

**Methods:**

We surveyed multiple clinical teams to determine drivers of diagnostic quality. Using the Model for Improvement, we implemented multiple interventions (clinical pathway revisions, visual guidance, direct provider feedback, and an improved light source option) in the care process for these patients and measured diagnostic quality using a definition adapted from a validated photograph-scoring system. We used statistical process control charts to track diagnostic quality.

**Results:**

We detected no special-cause variation in the outcome measure of improved diagnostic quality. We detected special-cause variation for the process measure of whether care teams obtained photodocumentation, which increased from 77% to 89%.

**Conclusions:**

Interventions to improve the diagnostic quality of anogenital photodocumentation did not result in improvement; however, there was improvement in the likelihood of care teams obtaining photodocumentation in acute sexual assault evaluations.

## INTRODUCTION

### Problem Description

More than 57,000 children were victims of sexual abuse in the United States in 2022.^1^ When concern for child sexual assault (CSA) arises, the care process often involves a forensic medical examination.^2^ This process can be costly,^3^ time-consuming,^4^ and a source of anxiety for children and families.^5^ Inefficiency is therefore wasteful and potentially harmful to victims.

### Available Knowledge

Whereas most children who undergo nonacute medical evaluations for sexual abuse have no abnormal findings on anogenital examination,^6,7^ in up to 14% of evaluations for CSA (generally defined as <72 h following an alleged incident), findings of penetrating trauma have been observed.^8^ Most genital injuries identified in children heal quickly without residual findings.^9,10^ Timely and accurate documentation of the anogenital examination is therefore of utmost importance.

Photodocumentation is part of the standard of care for children undergoing CSA evaluations.^2^ Providers often use telemedicine in forensic examinations, a practice that research has shown can increase the identification of clinical findings.^11^ Image quality is a key requirement for the accurate identification of genital findings. Poor quality contributes to errors in the evaluation of genital findings, which can result in improper care and erroneous reports to law enforcement and Child Protective Services.^12–14^

### Rationale

Photodocumentation in child abuse medical evaluations has several applications, including confirmation of findings, peer review, and use in court testimony.^15^ The National Children’s Alliance (NCA) recommends diagnostic-quality anogenital photodocumentation in all medical evaluations for suspected CSA.^16^ Despite this, NCA standards do not specify who should review photodocumentation or how they should assess quality, and there is no widely accepted practice standard for assessing anogenital photodocumentation quality in CSA evaluations. Although there is a validated assessment tool—the Photograph Documentation Image Quality Scoring System (PDIQSS)—its application has not been widely adopted.^17^ There is evidence demonstrating the benefits of peer review in child abuse evaluations with improvements in written and photodocumentation.^18^ Prior efforts using peer review to enhance quality in CSA evaluations showed improvement in the written and photodocumentation among pediatricians who conduct child abuse evaluations.^19^ However, no systematic approach has been published to improve the diagnostic quality of anogenital photodocumentation in CSA evaluations among emergency medicine providers and sexual assault nurse examiners, nor one that used a validated scoring system to assess photo quality.

### Specific Aims

Between July 1, 2022, and June 30, 2023, among sets of anogenital photodocumentation obtained for patients presenting to the Children’s Hospital of Philadelphia emergency department (ED) with concern for acute CSA, we found that 24% achieved diagnostic quality for accurate interpretation. The Children’s Hospital of Philadelphia Quality Improvement Framework SMART(IE) (Specific, Measurable, Achievable, Relevant, Time-based, Inclusive, and Equitable) aim was to improve the diagnostic quality of anogenital photodocumentation to 50% by March 31, 2024, regardless of patients’ racial and sociodemographic characteristics.

## METHODS

The project team used the Model for Improvement^20^ framework to develop and carry out this project and followed the SQUIRE 2.0 (Standards for Quality Improvement Reporting Excellence)^21^ guidelines for this report.

### Context

The project team conducted this project in the EDs at 2 campuses of a tertiary children’s hospital system that had more than 120,000 ED visits in FY 2023.^22^ The project team comprised child abuse pediatricians, advanced practice providers with expertise in CSA examinations, nursing leadership from the Sexual Assault Response Team (SART), and a pediatric ED physician. The SART program at this institution comprises 43 pediatric sexual assault nurse examiners who receive comprehensive education, including didactic instruction, clinical observation, simulation-based training, and guided mentorship. The SART program also provides education on forensic medical examinations to ED physicians. The ED initiates a SART activation for any pediatric patient presenting for acute sexual assault. A SART nurse or trained ED provider collects forensic evidence under the guidance of the SART team. CSA patients also undergo social work evaluations, reporting to Child Protective Services and/or law enforcement, medical examination, anogenital photodocumentation, testing and empiric treatment for STI and pregnancy, and/or HIV postexposure prophylaxis (PEP). Examining providers upload anogenital photodocumentation to the Epic (Epic Systems, Verona, WI) electronic medical record system.

The hospital’s Child Protection Team (CPT) is a subspecialty service that provides hospital consultations and may offer recommendations and care coordination, including a Child Abuse, Referral and Evaluation (CARE) Clinic visit. CPT members may provide remote, real-time assessments of photographs upon request by ED providers. CPT also reviews SART photographs when coordinating follow-up care for each CSA patient seen in the hospital’s ED; the absence of photographs, nondiagnostic photographs, or findings concerning for trauma are all indications to recommend CARE Clinic follow-up. Before this project, CPT did not routinely provide feedback on photograph quality to ED providers.

**Fig. 1.**
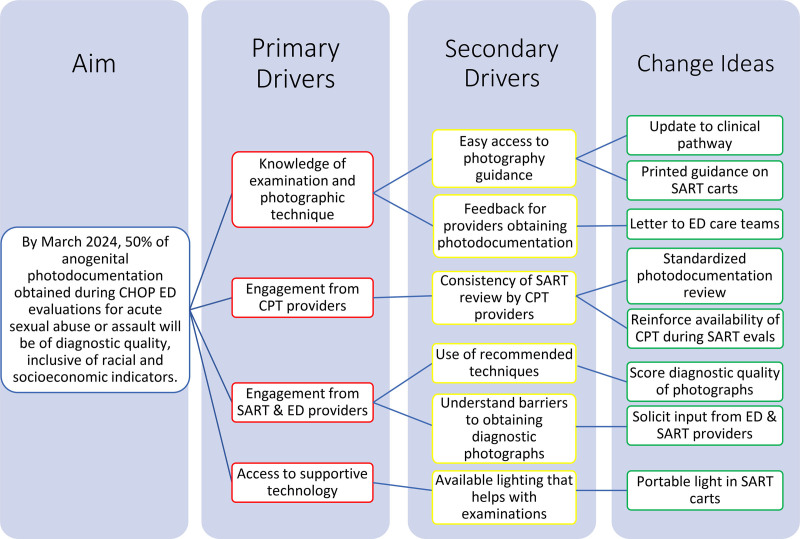
Driver diagram showing drivers of diagnostic-quality anogenital photodocumentation. CHOP, Children’s Hospital of Philadelphia.

To identify the root causes of poor photograph quality, we surveyed ED medical personnel, including physicians and advanced practice providers, about barriers to obtaining diagnostic photographs. We also surveyed CPT members on limitations to rendering diagnostic opinions from photographs. Figure 1 summarizes drivers of diagnostic quality.

### Interventions

From provider surveys, the project team developed a set of interventions that we implemented between July 2023 and February 2024 (Table 1). The team leader and project supervisor selected interventions for inclusion based on their potential impact on photodocumentation quality and the effort required.^23,24^

**Table 1.** Project Interventions and Dates of Implementation

	Intervention	Date Implemented
1	Updated hospital’s sexual abuse pathway with illustrated guidance on examination technique and photodocumentation technique based on review of existing literature^23,24^ and input from CPT and SART leadership (https://www.chop.edu/clinical-pathway/abuse-sexual-suspected-examination-and-photodocumentation-guidance)	July 2023
2	Single-page versions of pathway updates were affixed to forensic evidence collection carts at both ED campuses	July 2023
3	Revised CPT review of SART photographs to include assessment of barriers to diagnostic quality	September 2023
4	Feedback was emailed to ED care teams based on limitations identified by CPT in intervention 3 to support future improvements in photograph quality	September 2023
5	A clip-on light was added to the evidence collection cart at both sites. The light was selected for its ability to clip onto mobile phones of various widths and to optimize illumination of the phone’s rear-facing camera field of view. It was tested on an anogenital training manikin to confirm improved visualization	December 2023
6	Added information to the online pathway on the use of a clip-on phone light	February 2024

### Measures

Data collection and management were performed using REDCap electronic data capture tools hosted at the Children’s Hospital of Philadelphia.^25,26^ We selected charts based on their inclusion in the hospital’s SART database, a REDCap file that includes all patients for whom the ED activated a SART response. We collected demographic and clinical data from the SART database and medical records. We collected demographic data to determine whether we could identify differences in quality stratified by race, ethnicity, and indicators of socioeconomic status at baseline, and whether our interventions affected such differences. We considered a patient to have findings concerning for abuse based on the 2018 Adams criteria.^27^ The team lead assigned all anogenital photographs from the ED encounter to a project team member.

The primary outcome measure was the diagnostic quality of anogenital photodocumentation obtained during ED evaluations for acute CSA. To evaluate photograph quality, the project team used the PDIQSS, a validated scoring system developed to assess the quality of anogenital photodocumentation after sexual assault.^17^ Using a 16-point scale, reviewers scored all images from an ED encounter from 0 to 2 on 8 domains of image quality. Because PDIQSS is a tool for scoring image quality rather than diagnostic quality, the reviewers used the PDIQSS scoring process to standardize photograph assessment; however, we did not use the numerical score to determine whether the full set of photographs was of diagnostic quality. The project team established a priori consensus on the definition of diagnostic quality, with input from CPT physicians and SART leadership.

Diagnostic quality is defined by a set of photographs that

1. clearly shows the margins of the vulvar epithelium including the labia minora, the posterior fourchette, and the complete circumferential edges of the hymenal rim, as well as the anus for patients with female anatomy; or that clearly shows the ventral and dorsal aspects of the penile shaft, glans, and prepuce (if present) as well as the anus for patients with male anatomy;
2. displays the anatomic structures of the anogenital region in a way that allows for their clear identification and orientation in relation to one another.

After reviewing a set of photographs, the reviewer determined whether the complete set met this definition. Given the potential for bias to impact a reviewer’s assessment of diagnostic quality, the project lead blinded team members to whether the photographs were taken before or after the interventions. To this end, the project lead randomized and abstracted the charts nonchronologically. To evaluate interrater reliability, during the initial data collection period, second reviewers read 10% of the photograph sets from both baseline and intervention periods and made a new assessment of diagnostic quality using our a priori definition, with a resulting kappa of 0.34 for baseline cases, indicating minimal agreement, and 0.087 for intervention cases, indicating no agreement; neither met the minimum threshold of 0.40 to indicate adequate agreement. To address this, the team lead (who had not previously rated photographs) conducted a second review of every photograph set. Cases with discrepant assessments of diagnostic quality between the first review and the team lead’s review were reviewed again by at least 4 team members. Cases where team consensus differed from the initial review were recoded to the assessment of the consensus review team. Balancing measures included the percentage of patients who followed up in CARE Clinic, because CPT team members use photodocumentation quality for CARE Clinic referrals, and whether there was any disproportionality in diagnostic quality along sociodemographic characteristics.

### Analysis

We used a P-chart to track the primary outcome measure, the percentage of photograph sets achieving diagnostic quality, monthly for 1 year of baseline data and for 9 months after implementation of the first interventions. We also used a P-chart to track the process measure, the percentage of charts for which there was anogenital photodocumentation. We calculated measures comparing pre- and postintervention data using Stata Statistical Software.^28^

### Ethical Considerations

The hospital’s institutional review board reviewed the project plan and determined that it did not constitute human subjects research and therefore did not require ongoing institutional review board oversight. There were no financial conflicts of interest among project contributors.

## RESULTS

The study period analyzed data from July 1, 2022, to March 31, 2024, with initial interventions implemented on July 1, 2023, and final interventions implemented in February 2024, providing 1 year of baseline data, 9 months of data after initial interventions, and 2 months of data with all interventions. During the study period, there were 183 ED visits for acute CSA, 137 (75%) at the large urban site and 46 (25%) at the smaller suburban site (Table 2).

**Table 2.** Demographic Characteristics of Patients Who Presented to the ED for Child Sexual Assault During the Study Period, Overall and by Hospital Site

Demographic	All Patients, n = 183	Hospital Location: Philadelphia	Hospital Location: King of Prussia	χ^2^ (*P*)
Female sex, n (%)	151 (83)	114 (83)	37 (80)	0.18 (0.67)
Race/ethnicity, n (%)				18.3 (0.02)*
Black	97 (53)	83 (61)	14 (30)	
White	31 (17)	18 (13)	13 (28)	
Asian/Pacific Islander	2 (1)	1 (1)	1 (2)	
Hispanic	32 (17)	22 (16)	10 (22)	
Native American	1 (0.5)	1 (1)	0 (0)	
Unknown	20 (11)	12 (9)	8 (17)	
Insurance type, n (%)				3.1 (0.38)
Private	25 (14)	16 (12)	9 (20)	
Public	144 (79)	109 (80)	35 (76)	
Self-pay	10 (5)	8 (6)	2 (4)	
Unknown	4 (2)	4 (3)	0 (0)	
Zip code median income below national median, n (%)	158 (86)	126 (92)	32 (70)	14.7 (0.00)*
Zip code median income below national poverty line for family of 4, n (%)	62 (34)	61 (45)	1 (2)	27.6 (0.00)*

* *P* < 0.05.

Patient age had a mean of 11.3 years. Most demographics, including hospital location, sex, race/ethnicity, and estimated poverty (living in a zip code with a median income below the poverty line for a family of 4),^29,30^ did not differ significantly between the pre- and postintervention groups.

Estimated poverty was significantly higher among patients presenting to the urban site. Of note, there was a significant difference in insurance types between the pre- and postintervention groups (χ^2^ = 20.03, *P* < 0.05), with a postintervention decrease in patients on public insurance and increases in patients on private insurance and self-pay.

**Fig. 2.**
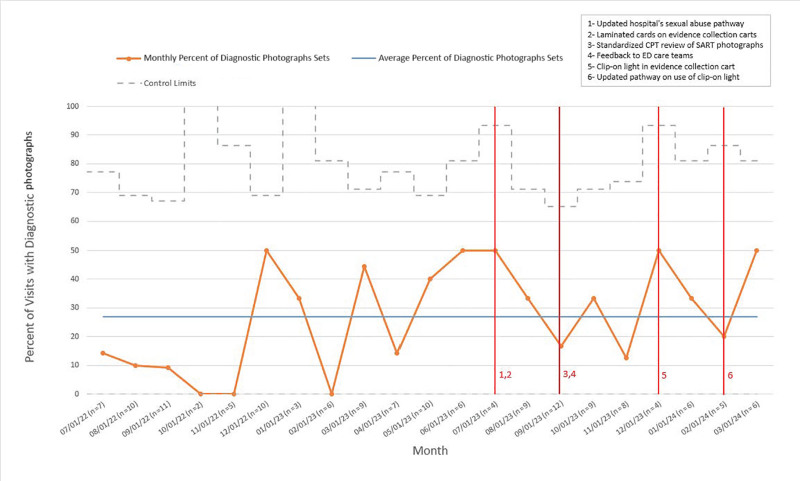
Statistical process control chart showing the monthly rate of diagnostic-quality photodocumentation with the timing of interventions.

**Fig. 3.**
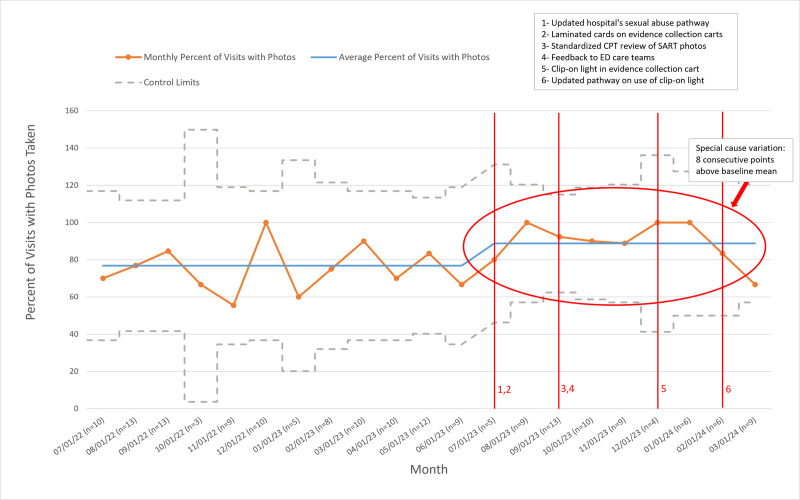
Statistical process control chart showing the rate of charts for which photodocumentation was obtained with the timing of interventions and special-cause variation highlighted.

We used statistical process control charts and standard rules to assess the effect of our interventions, using special-cause variation to define the charts. We did not identify special-cause variation in the primary outcome measure (Fig. 2), indicating that interventions did not result in a detectable change in diagnostic quality. We detected special-cause variation in the primary process measure, the percent of patients for whom anogenital photodocumentation was obtained (Fig. 3).

Although there was no significant increase in the number of photographs obtained per patient postintervention (*t* = −1.94, *P* = 0.054), a higher number of photographs obtained was associated with improved diagnostic quality (odds ratio [OR] = 1.27; 95% CI 1.10–1.48). Excluding cases without photodocumentation, there was no higher number of photographs per case postintervention (OR 1.08; 95% CI 0.96–1.21), which may help explain the lack of a detectable increase in diagnostic quality. Patients who had photographs taken were more likely to have evidence collection (χ^2^ = 29.4, *P* = 0.00) and to be scheduled for CARE Clinic (χ^2^ = 9.77, *P* = 0.002).

There was no significant change in whether patients were scheduled for or attended CARE Clinic postintervention. Patients who received an HIV PEP prescription as part of their ED care were more likely to follow up in CARE Clinic than those who did not (χ^2^ = 41.4, *P* = 0.00). Estimated poverty was associated with a lower likelihood of having diagnostic-quality photographs taken (χ^2^ = 4.51, *P* = 0.034), though it did not correlate with fewer photographs (OR 0.95; 95% CI 0.85–1.05). Patients were more likely to undergo forensic evidence collection in the postintervention period (*P* = 0.048).

The anatomical regions highlighted in the photographs were a factor in the assessment of diagnostic quality: of 149 photograph sets, 38 (25%) captured only the genital region, 3 (2%) only the anal region, and 108 (72%) captured both. Thirty-seven percent of photograph sets capturing both regions were of diagnostic quality. There was no change in which regions were highlighted postintervention (χ^2^ = 5.06, *P* = 0.08).

## DISCUSSION

### Summary

Interventions to improve the diagnostic quality of anogenital photodocumentation in CSA evaluations did not achieve the primary aim, as evidenced by the lack of special-cause variation in the primary outcome (Fig. 2). Special-cause variation was identified in a process measure (Fig. 3), showing an average 12% postintervention increase in the proportion of patients with photodocumentation. Additionally, we found a significant association between a higher number of photographs obtained and diagnostic quality.

### Interpretation

There are multiple possible reasons that we did not achieve our primary aim. It may be that the interventions were not effective at improving photograph quality, even though we designed them to address the root causes of poor quality. On-site guidance on examination techniques from CPT members would likely have a greater impact than, for example, retrospective feedback. It may be that the process of rating photograph quality would have failed to detect improvement, though the use of a validated scoring system and an expert consensus model to define diagnostic quality should ideally have prevented this. The intervention period may have left insufficient time for improvements to become measurable; having only 2 months of data once all interventions were in place supports this possibility. The success of the process measure—to increase the percent of visits with photodocumentation—indicates that interventions did result in measurable change in ED providers’ practice. This result is likely attributable to the emphasis on photodocumentation in the updated clinical pathway.

We identified additional significant findings in the review of project data. A larger number of photographs significantly increased the likelihood that a photograph set would attain diagnostic quality. This finding indicates that clinical guidelines on anogenital photodocumentation should include a recommendation to obtain multiple photographs. Having photographs taken was associated with a higher likelihood of being scheduled but not seen for clinic follow-up; it may be that patients who had photographs taken experienced barriers to follow-up care that we did not collect data on, such as a reluctance to undergo repeated examination. The association between being prescribed HIV PEP with clinic follow-up is likely attributable to the fact that patients on PEP are routinely scheduled for the CARE Clinic to coincide with monitoring tests. Intervention patients being more likely to have private insurance or self-pay rather than public insurance compared with baseline may be related to a change in Pennsylvania’s Medicaid renewal policy implemented in April 2023, which coincided with a statewide decrease in Medicaid enrollment.^31^ The increase in evidence collection postintervention may suggest that providers modified their care for CSA patients because they were aware of an ongoing QI project. Another notable observation was that estimated poverty correlated with poorer photograph quality (Table 3). Multiple possible factors may have played a role in this finding, which could be provider-related (subconscious bias leading to decreased time or effort with such patients) or patient-related (increased anxiety, communication barriers, health comorbidities). The health equity implications of this finding warrant further study to identify facilitators and barriers that may lead to differential quality of diagnostic information along income lines. Notably, there was no association found between diagnostic quality and race/ethnicity (χ^2^ = 1.48, *P* = 0.98) or insurance type (χ^2^ = 5.3, *P* = 0.15).

**Table 3.** Association of Patient Demographics With Outcome Measure of Diagnostic Quality Among Patients for Whom Photographs Were Taken

Demographic	Photographs Were of Diagnostic Quality (n = 40, 27% of Total)	Photographs Were Nondiagnostic (n = 109, 73% of Total)	χ^2^ (*P*)
Hospital location, n (%)			0.52 (0.47)
Philadelphia	32 (28)	81 (72)	
King of Prussia	8 (22)	28 (78)	
Female sex, n (%)	36 (29)	87 (71)	2.1 (0.15)
Race/ethnicity, n (%)			1.48 (0.98)
Black	22 (29)	55 (71)	
White	6 (24)	19 (76)	
Asian/Pacific Islander	0 (0)	2 (100)	
Hispanic	7 (25)	21 (75)	
Native American	0 (0)	0 (0)	
Unknown	5 (29)	12 (71)	
Insurance type, n (%)			5.3 (0.15)
Private	10 (45)	12 (55)	
Public	28 (25)	86 (75)	
Self-pay	1 (11)	8 (89)	
Unknown	1 (25)	3 (75)	
Zip code median income below national median, n (%)	36 (28)	92 (72)	0.76 (0.384)
Zip code median income below national poverty line for family of 4, n (%)	8 (16)	42 (84)	4.51 (0.034)*

* *P* < 0.05.

### Limitations

There are several limitations in the implementation and data collection for this project. Because the project lead randomized chart reviews to blind reviewers, we reviewed some charts months after interventions were implemented, limiting their usefulness in supporting the iterative nature of PDSA cycles. The reason we chose this methodology is described in the Methods section. Second, the data collection process required revision after we identified poor interrater reliability, which the team lead may have mitigated through quality checks to ensure that reviewers applied the definition of diagnostic quality consistently. The project team revised their approach and successfully reached consensus on each photograph set that required review; however, we acknowledge that this review process, including the lack of further reliability testing on the consensus-reviewed photograph sets, risked introducing bias in arriving at consensus for each case. It is also possible that the initial poor interrater reliability and subsequent review process negatively impacted our ability to detect special-cause variation. Third, the small number of photograph sets obtained per month may have limited the ability to detect change, and a longer intervention phase may have allowed more time for improvement to occur and to be detected. Fourth, the fact that there is no widely used practice standard for photodocumentation of the male genital examination limited the definition of diagnostic quality, and the project’s definition may have been more restrictive than what providers use in common practice; a less restrictive definition may have shown a higher likelihood of diagnostic quality for patients with male anatomy.

## CONCLUSIONS

Although we did not achieve the goal of improving the diagnostic quality of anogenital photodocumentation in the evaluation of acute CSA, this project did demonstrate improvements in obtaining photodocumentation, with more images associated with higher diagnostic quality. The project’s findings highlight opportunities for future efforts. The need to identify a photograph-scoring system and to define photograph quality highlights the lack of a practice standard for anogenital photodocumentation assessment, despite the requirement for peer review for NCA accreditation.^16^ The project team demonstrated, through its work, the benefit of multidisciplinary analysis of photograph imaging. Anecdotally, numerous providers expressed appreciation to the team leader regarding the practice of providing direct feedback on photograph quality, which could be applicable in settings where child abuse pediatricians and forensic nurses are not always providing on-site care. The use of a clip-on light for photographs taken with smartphones is an easily implemented technology in the medical care of sexual abuse victims.

## ACKNOWLEDGMENTS

The project team wishes to acknowledge E.J. Ernst, DNP; Patricia M. Speck, DNSc; and Joyce J. Fitzpatrick, PhD, for providing insight into the PDIQSS, including access to information about the tool’s development and clinical use. The project team also wishes to acknowledge Jane Lavelle, MD, for supporting the project’s development and implementation. Dr. Miller wishes to acknowledge the faculty of the Perelman School of Medicine’s Center for Healthcare Improvement and Patient Safety for their guidance.
